# (Micro)Plastics Are Toxic Pollutants

**DOI:** 10.3390/toxics11110935

**Published:** 2023-11-17

**Authors:** Judith S. Weis, Juan José Alava

**Affiliations:** 1 Department of Biological Sciences, Rutgers University, Newark, NJ 07102, USA; 2 Ocean Pollution Research Unit & Nippon Foundation-Ocean Litter Project, Institute for the Oceans and Fisheries, University of British Columbia, Vancouver, BC V6T1Z4, Canada; j.alava@oceans.ubc.ca

**Keywords:** plastics, microplastics, bioaccumulation, toxicity, sorption, leaching, biofilm

## Abstract

Plastics, including microplastics, have generally been regarded as harmful to organisms because of their physical characteristics. There has recently been a call to understand and regard them as persistent, bioaccumulative, and toxic. This review elaborates on the reasons that microplastics in particular should be considered as “toxic pollutants”. This view is supported by research demonstrating that they contain toxic chemicals within their structure and also adsorb additional chemicals, including polychlorinated biphenyls (PCBs), pesticides, metals, and polycyclic aromatic hydrocarbons (PAHs), from the environment. Furthermore, these chemicals can be released into tissues of animals that consume microplastics and can be responsible for the harmful effects observed on biological processes such as development, physiology, gene expression, and behavior. Leachates, weathering, and biofilm play important roles in the interactions between microplastics and biota. Global policy efforts by the United Nations Environmental Assembly via the international legally binding treaty to address global plastic pollution should consider the designation of harmful plastics (e.g., microplastics) with associated hazardous chemicals as toxic pollutants.

## 1. Microplastics Occurrence

**Figure 1:**
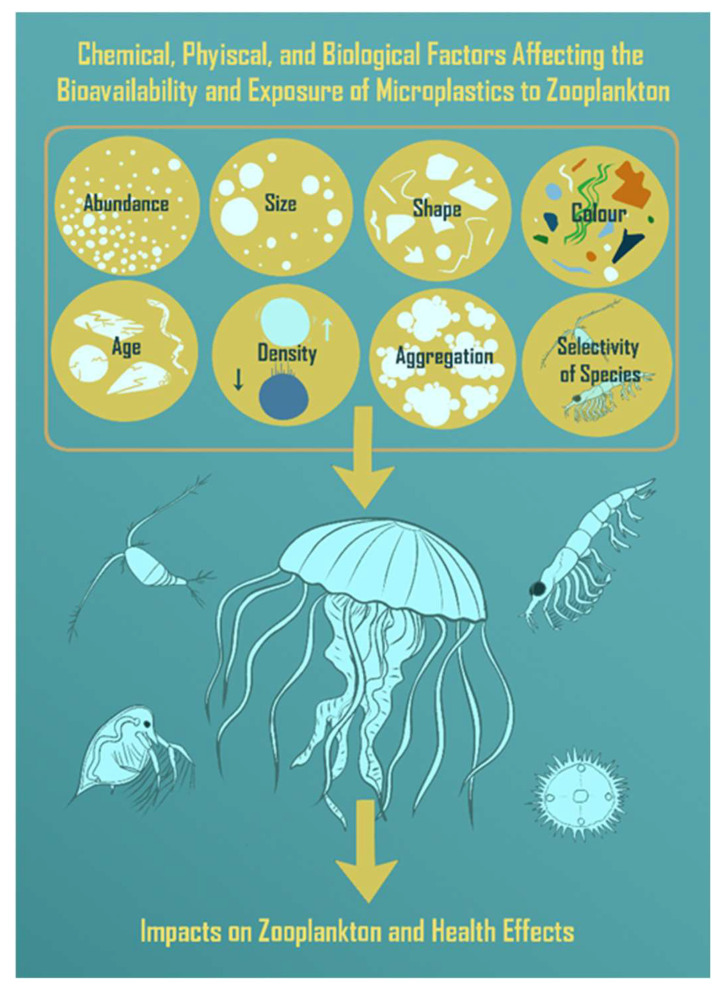
Diversity of microplastic particles (MPs) with bioavailability as a function of their physical–chemical properties, influencing exposure to zooplankton. Based on the review by Botterell et al. (2019) [1].

Microplastics (MPs, with a particle size from 1 µm to <5 mm) are ubiquitous pollutants on earth; these emerging pollutants have been found everywhere that they have been looked for in terrestrial and in aquatic (marine and freshwater) systems, as well as in the air (atmospheric transport and deposition). They are diverse since they can be made of many different polymers, have a variety of different chemical additives, and come in many shapes, sizes, and colors, affecting aquatic organisms (Figure 1) [1]. These plastic particles are readily taken up into organisms via ingestion and respiration and are found throughout food webs. Their effects have been studied extensively in a wide range of plants and animals; health effects ranging from genetic and biochemical up to organismal levels have been reported [1,2].

Some of their observed negative impacts (i.e., tissue injury) can be tied to physical effects caused by their shape, size, volume, density, and roughness, while other impacts are due to the toxicity of chemical additives that can leach from the plastic (e.g., bisphenol A (BPA), phthalates, polybrominated diphenyl ethers (PBDEs), and per- and polyfluoroalkyl substances (PFASs), among other toxic substances) [2,3,4]. In addition, MPs that have been in the environment for a period of time get “weathered” and acquire additional chemicals adsorbed from the environment [5,6]. In this paper, we focus on the toxic chemicals associated with MPs (both in and on them) and environmental effects that can be attributed to them. A Google Scholar search was followed by a study of references in relevant review articles.

## 2. Chemicals in MPs including Microfibers (MFs)

Plastics are polymers created from monomers. Additional chemicals are added to give the plastic particular characteristics. Some of these are carcinogens or endocrine disruptors such as bisphenol A and phthalates (plasticizers). According to the UNEP [3] and Wiesinger et al. (2021) [7] assessing plastic and additives on the global market, there are over 13,000 different chemicals identified, of which over 3200 are considered substances of potential concern (persistent, bioaccumulative, or toxic). Many of these chemicals have not been studied, so their toxicity is unknown. Some toxic chemicals that are used in the production of MPs include perfluorooctanesulfonic acid (PFOS). Bisphenol A (BPA) and phthalates are known endocrine-disrupting chemicals that affect development and reproduction in humans and other species [8,9]. BPA and phthalates from MPs may also induce changes in the neuroendocrine system and signaling [10,11]. BPA can pass through the blood–brain barrier, and exposure is linked with neuropsychological dysfunction, neurobehavioral disorders, and neurodegenerative disease [12], as well as affecting DNA methylation [13,14] and epigenetic alterations that impact heart development and metabolism [15,16]. 

Tire wear particles (TWPs) are a type of rubber and microplastic that are very common in the environment and are especially toxic to salmonid fishes. Mortalities of coho salmon (*Oncorhynchus kisutch*) were linked to environmental levels of toxic chemicals used as antiozonant (e.g., 6PPD and 6PPD-quinone, which reduce damage from ozone) from TWPs in the U.S. Pacific Northwest [17]. 

The largest percentage of microplastics in the terrestrial and aquatic environment are microfibers (MFs), which are released primarily from synthetic clothing when manufactured, worn, disposed of, or in washing machines or dryers [18]. Textile MFs contain a great variety of other chemicals including dyes, antimicrobial agents, wrinkle resistance, finishing, and water and stain repellents [19], many of which can affect human and environmental health [20]. The largest group of chemicals in textiles are pigments and dyes. The health effects of these chemicals can include allergic reactions, carcinogenesis, and effects on growth and development [21]. 

Other sources of MFs include carpeting and personal care products [22,23,24]. Cigarette filters, made of cellulose acetate fibers (semi-synthetic plastic) [25], are another major source of MFs. It has been estimated that 4.5 trillion cigarette filters are littered annually, generating about 0.3 million tons of MFs annually [26]. Toxic chemicals that are associated with cigarette filters (butts) include polycyclic aromatic hydrocarbons (PAHs) and metals. Leachates from cigarette filters have been shown to be toxic [27,28,29]. Belzagui et al. (2021) [25] found that the negative effects of cigarette leachates are intensified by the physical effects of the fibers. An additional significant source of microfibers is fishing gear (nets and ropes), including lost and abandoned gear (“ghost” gear [30]).

## 3. MPs Accumulate Toxics from the Environment

Microplastics exhibit low polarity on their surface, which facilitates the hydrophobic adsorption of chemicals from seawater [31]. This adsorption is affected by weathering (changes in surface morphology and fragmentation) and the type of plastic. For example, there is greater sorption of polychlorinated biphenyls (PCBs) to PE (polyethylene) pellets than to PP (polypropylene) pellets. The low polarity of MPs could also be responsible for metal ion adsorption on their surfaces, since metal ions are positively charged and bind to negatively charged groups on the plastic surface [31].

MPs have a high surface area-to-volume ratio, making them a good sorbent for toxic chemicals [32]. The sorption/desorption of a chemical depends on its physical–chemical properties as well as the physical/chemical properties of the MPs. MPs including MFs from textiles adsorb chemicals, including metals, pharmaceuticals, PAHs, and PCBs [31,33,34,35]. While most research on the sorption of chemicals to MPs has focused on spheres and fragments [36], the sorption and desorption of chemicals from textile fibers [37,38] have also been demonstrated. 

The weathering of the MP can change its sorption ability. MPs (including MFs) degrade in the environment via photodegradation. The degree of weathering varies among different types of MPs and different chemicals (Sait et al., 2021) [37], in both new and weathered MPs. Associated chemicals also differ between virgin vs. weathered MPs, including fibers [38,39]. 

### 3.1. Metal Contaminants

High concentrations of environmental metals attach onto MPs. Rochman et al. (2014) [40] studied metal accumulation on new (virgin) MPs, including polyethylene terephthalate (PET), high-density polyethylene (HDPE), polyvinyl chloride (PVC), low-density polyethylene (LDPE), and polypropylene (PP), in San Diego Bay, USA, and average concentrations on all polymers after one year were highest for Zn and lowest for Pb. Munier and Bendell (2018), and Kazmiruk (2023) [35,41] studied Cd, Cu, Pb, and Zn sorbed on different MPs (PVC, nylon, polyethylene terephthalate (PET), polystyrene (PS), low-density polyethylene (LDPE), high-density polyethylene (HDPE), polycarbonate (PC), polyester (PE), and polyurethane (PUR)) in urban intertidal regions in Vancouver, BC, Canada. LDPE generally adsorbed higher concentrations of all the metals than other polymers. 

### 3.2. Organic Contaminants

PCBs are found worldwide attached to MPs. Frias et al. (2010) [42] found concentrations of 0.02–15.56 ng/g on MPs from Portugal beaches. Hirai et al. [43] found 1–1000 ng/g PCBs from MPs from open oceans, and both remote and urban beaches. Plastic pellets from remote islands in the Pacific, Atlantic and Indian Oceans and the Caribbean Sea had PCB concentrations of 0.1–9.9 ng/g [44].

PAHs are also found worldwide. Rios et al. (2007) [45] collected MPs from North Pacific Gyre, California, Hawaii, and Mexico, and reported adsorbed PAHs of 39–1200 ng/g. They also noted that yellowed (more weathered) plastic had higher concentrations (6100–12,000 ng/g) [45]. Samples from the bank of the San Gabriel River, the beach, and the sea surface also had high (6200–9200 ng/g) PAHs. Total PAHs in pellets from Kato Achaia, Greece, had 66- 637 ng/g [46]. Lower concentrations (0.2–319.2 ng/g) were found in MPs on two Portuguese beaches [42]. Sao Torpes Beach, Sines in Portugal (24,400 ng/g-pellet) and Forth Estuary in the UK (164,900 ng/g-pellet) had the highest PAH concentrations from 75 locations in 26 countries, which was attributed to crude oil pollution [42]. Plastic pellets from a beach near an oil refinery in Ghana were also highly contaminated, with 2751 ng/g-pellet [47], confirming the effects of crude oil pollution.

### 3.3. Pesticides

The concentrations of DDT and similar compounds vary by location and methodology. Ogata et al. (2009) [48] analyzed plastic pellets from 30 beaches from 17 countries and five continents (North America, Europe, Asia, Africa, and Oceania) for DDT, DDD, and DDE and found the highest concentrations of total DDTs at Hermosa Beach, California, USA (267 ng/g), while the Bay of Maputo, Mozambique, and South Durban, South Africa, had concentrations of 4.49 ng/g and 2.43 ng/g, respectively. Colabuono et al. (2010) [49] analyzed MPs ingested by seabirds, and found total DDT of 64.4–87.7 ng/g. Among the 25 different organochlorine pesticides studied, p,p’-DDE had the highest concentrations. This study also analyzed MPs in the digestive tract of eight species of *Procellariiform* birds in Brazil and found amounts of total chlordane of 4.29–14.4 ng/g, total cyclodienes of 2.41–50.9 ng/g, total mirex from 6.48 to 14.6 ng/g, and total hexachlorobenzene from 12.4 to 17.5 ng/g. 

### 3.4. PFAS

Some consumer products (textiles, carpets, and plastics) are known to be sources of both PFAS and MPs through their manufacture, use, and cleaning, resulting in MP-PFAS complexes in the environment [50]. Since hydrophobic interactions are important for sorption behaviors, and long-chain PFAS are more hydrophobic, they are more likely to adsorb than short-chain PFAS compounds as observed on beached plastic pellets from Greek islands, which had a concentration range of 10–180 ng/kg [51].

## 4. The Role of Biofilm in Affecting the Transfer of Contaminants onto and from MPs

As MPs age in the environment, they acquire a biofilm consisting of a community of microbes. The weathering of MPs [52] alters contaminant adsorption and incorporation into the biofilm (“plastisphere”). Plastic particles floating or settling interact with contaminants, which influence their transport. Peng et al. (2023) [53] found metal loss from plastics submerged in coastal seawater for eight months and studied the role of biofilm in controlling the leaching of Sb, Sn, Pb, Ba, and Cr. They found that warmer temperatures promoted the release of the metals. UV radiation significantly increased the leaching of Sn from polylactide (PLA), and high salinity promoted the leaching of Sn from PLA and Pb from polyvinylchloride but inhibited the leaching of Ba from PE. Metal loss from the plastics in the field took place rapidly during the first three weeks but then was slowed by the development of biofilm. The characteristics of the formation of biofilm on MPs, including sizes, types, and degradability, environmental conditions (e.g., nutrients, pH, and dissolved organic matter), biofilm maturity, and metal species, all influence metal adsorption–desorption by plastic particles [54]. In comparison to bare MPs, metal adsorption capacity is increased by the biofilm, mainly attributed to the strengthening of electrostatic interactions, coordination, complexation, and surface precipitation, as well as additional ion exchange [54]. In addition, charged organic contaminants can cause MPs to aggregate in the water and even form flocs [55].

## 5. Chemicals Transfer from MPs into Organisms 

**Figure 2:**
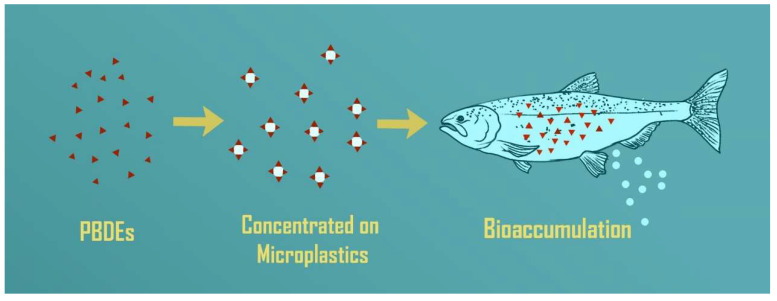
Illustration showing persistent organic pollutant sorption and accumulation over time, using flame-retardant compounds (polybrominated diphenyl ethers (PBDEs)) as example, via microplastic ingestion in fish. Adapted from Wardrop et al., 2016 [57]. 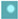 = microplastics (MPs); 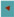 = chemical contaminant or pollutant; and 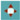 = microplastic with sorbed pollutant.

Organisms take up chemicals from ingested particles depending on gut residence time, which varies among different types and shapes of microplastics and the length, complexity, and enzymes of the digestive system of the organism. Another important consideration is the bioavailability of sorbed contaminants from MPs vs. other sources. [56,57,58]. Wardrop et al. [59] investigated whether PBDEs sorbed onto MPs were assimilated by fish following ingestion. Experimental rainbow fish (*Melanotaenia fluviatilis*) had significantly higher PBDE concentrations than controls after 21 d (Figure 2). Lower brominated congeners had the highest accumulation, and longer exposure time resulted in increased accumulation. Herrera et al. (2022) [60] fed European seabass (*Dicentrarchus labrax*) for 60 days with three treatments: control (feed), feed with 10% virgin microplastics, and feed with 10% environmental microplastics. Additives such as PBDEs and chemicals adsorbed from the environment such as PCBs were analyzed in the environmental MPs, feed, and liver. The results showed that both additives and contaminants on the environmental MPs bioaccumulated in the fish liver after ingesting the MPs. 

In contrast, Beckingham and Ghosh (2017) [61] determined that PCB transfer from MP spheres into benthic worms was much less than transfer from surrounding sediments. Thaysen et al. (2020) [62] found the bidirectional transfer of PBDEs from ingested microplastics in seabirds; contaminants in tissues could transfer onto ingested microplastics. Given the diversity of MPs and their associated chemicals, generalizations are not possible. 

Since results on the desorption of chemicals from MPs are not consistent, it is not possible to conclude whether or not POPs desorbed from MPs are always harmful. Negative findings may be due to differences among polymer types and plastic properties, the physicochemical properties of the toxicant (e.g., octanol–water partition coefficient (K_OW_) and molecular weight), exposure routes (water, ingestion, and inhalation), and species [63,64]). 

## 6. The Effects of Aging, Weathering, and Leachates

MP aging involves photodegradation, biodegradation, and mechanical fragmentation, which affect interactions with environmental contaminants. The rate and extent of MP aging are affected by their physicochemical properties in addition to the environmental factors that determine the adsorption of environmental chemicals onto aged MPs. Metals and organic contaminants tend to accumulate on MPs via adsorption, and the interactions between them impact their environmental behavior [64]. Aging enhances the specific surface area and functional groups of MPs, thereby affecting interactions with contaminants.

There is a huge amount of research in the literature in which experimental organisms are exposed to MPs and various effects are documented on biochemistry, gene expression, physiology, growth, development, reproduction, behavior, etc. Most of these studies cannot distinguish whether the effects are due to the physical presence of the particles or to chemical toxicity. There are some experimental approaches that allow this distinction to be made. Comparing the toxicity of virgin versus weathered plastics is one way to do so. These studies generally indicate that weathered particles (which have adsorbed chemicals) are more toxic than virgin particles [65,66,67]. Rios-Fuster et al. (2021) [68] exposed *Sparus aurata* juveniles for 21 days to both virgin and weathered MPs, then examined enzyme biomarkers and social and feeding behavior. They observed increased cellular stress from virgin MPs, and a much larger response from the weathered MPs. After exposure to weathered MPs, fish showed greater boldness during social interactions than controls. Some other studies have shown aged MPs to have lower toxicity, however [69]. Weathered MPs may, thus, produce a greater response than virgin MPs, but the opposite may also be true, owing to the complex process of biofouling and chemical leaching and the identity and concentration of the chemicals that adsorbed on the weathered MPs. 

Another approach is to spike the MPs with selected chemicals and compare the responses of organisms exposed to “virgin” vs. spiked MPs. Le Bihanic et al. (2020) [5] exposed marine medaka embryos (*Oryzias melastigma*) to MPs spiked with benzo(a)pyrene (BaP), perfluorooctanesulfonic acid (PFOS), or benzophenone-3 (BP3). They observed that the MPs agglomerated on the outer membrane (chorion) of the eggs and did not go through it. While embryos treated with virgin MPs were normal, those exposed to MPs with BaP or BP3 had reduced growth, developmental abnormalities, and abnormal behavior; those treated with MPs with PFOS had reduced survival and did not hatch. Thus, only the chemicals penetrated the chorion, and they had chemical-specific effects.

The toxicity of leachates may depend on weathering processes such as mechanical abrasion and photochemical oxidation [70]. Fries and Suhring (2023) [71] found 52 plastic additives were leached in a leaching protocol, of which 44 were unique chemicals based on mass and retention time. The greatest numbers of plastic additives were leached when the plastic was in lake water, was exposed to UV light, and had a longer time (about 30 days) for leaching. 

## 7. Toxicity Studies on MP Leachates

Most toxicity studies on MPs expose the organisms to some concentration of some type of MP and report on the effects produced. This experimental design cannot distinguish whether the effects were due to the physical effects of the MPs or to the chemicals they transferred to the organisms. In addition to comparing weathered vs. virgin MPs or studying spiked MPs, described above, another way to see if the effects are due to toxicants is to investigate leachates. Numerous studies have been conducted with leachates that demonstrate effects on mortality, reproduction, development, behavior, and other processes in a wide variety of species. Mortality was seen in barnacle larvae (*Amphibalanus amphitrite)* exposed to PC, HDPE, LDPE, PET, PP, PS, and PVC leachates [72]. Mortality was seen in adult mussels exposed to PP, PVC, car tire rubber (CTR), and leachates from tires (*Mytilus galloprovincialis)* [73]. The mortality of adult copepods *Nitocra spinipes* increased after exposure to leachates of PUR, aged polyisocyanurate rigid (foam) (PIR), and bio-PET particles [74]. In contrast, no significant mortality was observed with PE, PET, PP leachates, or PLA and bio-PET leachates. The mortality of adult copepods was not affected by LDPE leachates, indicating that the survival of adult copepods exposed to leachates depends on the polymer type and the species.

Plastic leachate’s effects on reproduction include the egg production, fertilization, hatching, and larval settlement of various taxa. The settlement of barnacle nauplii *A. amphitrite* decreased in many polymer types [72]. Exposure to virgin and aged car tire rubber (CTR) leachates did not affect egg production or hatching of the copepods *Acartia tonsa* and *Temora longicornis*, while exposure to TWP leachate increased *A. tonsa* egg production but not hatching [74,75]. No changes in fertilization were observed in the sea urchin *Paracentrotus lividus* exposed to polymethyl methacrylate (PMMA) or PS leachates [76] However, exposure to PET, PS, PVC, PP, and CTR leachates decreased the fertilization of the mussel *M. galloprovincialis* [73]. Thus, the effects of plastic leachates depend on the polymer type and test species. The concentrations and identities of different chemicals in the various leachates are generally unknown.

The impacts of MP leachates on embryonic development have been studied in many taxa. PE leachate induced abnormal embryonic development in the sea urchins *Lytechinus variegatus* [77] and *P. lividus* [78]. The embryonic development of *P. lividus* was impaired by leachate from PET [79], PVC [80], and beached pellets mainly composed of PE [80]. However, similar concentrations of leachate from beached pellets did not affect the development of *L. variegatus* embryos [77], suggesting that toxicity depends on the cocktail of additives and pollutants that are released during the leaching process. The effects of virgin polyvinyl chloride (PVC) microspheres and their leachates on the embryo-larval development of sea cucumber *Apostichopus japonicus* were dose- and time-dependent, but the toxicity of the leachates was greater than that of the microspheres [81]. Similar abnormal development, including abnormal gastrulation, was seen in embryos exposed directly to PVC. Chemical analyses of PVC microspheres and leachates revealed five phthalate esters, with DIBP (diisobutyl phthalate) and DBP (dibutyl phthalate) being high in the PVC leachates, suggesting that the elevated toxicity of leachate may be due to the leaching of phthalates. Developmental abnormalities were observed in the mussel *M. galloprovincialis* exposed to PET, PS, PVC, PP, and CTR leachates [73]. The Le Bihanic et al. (2020) [5] study cited previously found that different chemicals spiked on MPs had chemical-specific developmental effects, while the MPs themselves did not penetrate the outer membrane. This study indicates there is a transfer of toxicants from MPs through the chorion of fish embryos.

Behavior has been shown to be affected in various taxa. The aggregation of mussels, specifically the percentage of aggregated mussels, time to aggregate, and the crawling distance, increased with exposure to PP leachate for *Choromytilus meridionalis* and *Mytilus edulis* [82]. However, the reverse was seen in *M. galloprovincialis* and *Perna perna* [82]. Seuront (2018) [67] treated periwinkle snails (*Littorina littorea*) with leachates from virgin and beached pellets. The virgin pellets impaired vigilance and antipredator behavior slightly; however, beached pellets with adsorbed pollutants inhibited these behaviors severely. These behaviors are important for predator avoidance. Ricorte et al. (2021) [83] exposed the larvae of zebrafish, *Danio rerio* (which is not especially sensitive to 6PPD-quinone), to three environmental levels (i.e., 20, 200, and 2000 ng/L) of 6PPD-quinone (from tires) for 24 h, then examined the effects on exploratory behavior, the escape response, learning, neurotransmitters, the wake/sleep cycle, and circadian rhythm. Exposure to the two lowest concentrations caused changes in exploratory behavior and learning (habituation), which were consistent with the observed increased levels of neurotransmitters (acetylcholine, norepinephrine, epinephrine, and serotonin). Exposure to the highest concentration altered the wake/sleep cycle and expression of circadian clock genes. 

## 8. Discussion 

In the “Plasticene” or the Age of Plastics [84], environmental pollution by plastics, including large plastics and plastic particles, is one the most pervasive ecological footprints generated by anthropogenic activities and presents continual stress, reshaping biogeochemical cycles and threatening the planetary boundaries of the Earth [85,86]. The chronic impacts and health risks due to the accelerated rate of plastic emissions and contamination of the global ocean environment and coastal zones are unprecedented. MPs, in tandem with associated toxic chemical additives, are of great concern in the global ocean, freshwater, and terrestrial environments. The weight of evidence from the existing literature in this review demonstrates that chemical toxicity causes deleterious effects in aquatic organisms after plastic particle exposure, ingestion, and accumulation [87]. 

While environmental MP concentrations range in the order of <1 ng/L to ~1 μg/L [85] or <1–500 MP particles/m^3^, with a particle diameter of >5–500 µm [17,88], concentrations used in lab studies of MPs can be orders of magnitude higher, including in studies of plastic leachate. Effects seen in various leachate studies cannot be directly compared with one another since the range of concentrations, the nature of the polymers and additives in leachate solutions, as well as the leaching time and exposure time differ greatly among experiments. Also, many studies of MPs themselves use shapes (microbeads or spheres) that do not reflect the types that are predominant in the environment (microfibers) or exposure times [88,89]. Environmentally realistic concentrations should be used in leachate (and all MP) studies to better understand the impacts and ecological consequences of microplastic contamination.

## 9. Conclusions

In a short viewpoint paper, Alava et al. [90] argued that harmful plastics, including MPs, should be considered PBT (persistent, bioaccumulative, and toxic) chemical pollutants. Expanding upon that, this review of the literature is designed to highlight that MPs (1) contain toxic chemicals, including bisphenols and phthalates that are carcinogens or endocrine disruptors, and (2) MPs can adsorb additional chemicals from the aquatic environment, including metals, PAHs, chlorinated pesticides, and industrial chemicals such as PCBs. Studies have also demonstrated that (3) these chemicals can be transferred to exposed organisms and (4) chemicals from plastics can cause toxic effects. Experimental studies of chemical toxicity can be performed by comparing virgin vs. weathered MPs, by using MPs “spiked” with specific chemicals, or by using leachates. Such studies have demonstrated a variety of deleterious effects on development, reproduction, behavior, growth, etc., that appear to be related to the particular chemicals involved. For results to be meaningful and relevant, and the field to progress, researchers are encouraged to use environmentally relevant concentrations and types of MPs in exposure studies. 

In March 2022, the United Nations Environmental Assembly (UNEA) adopted a resolution to develop an international legally binding treaty to address global plastic pollution [91]. Within the precautionary approach, based on the best available toxicological science for microplastics, it is vital that the toxic aspects of plastics, including MPs, be included in this important treaty.

## Data Availability

Additional data is available upon request to the first author (J.S.W.).
